# Material Analysis of Steel Fibre Reinforced High-Strength Concrete in Terms of Flexural Behaviour. Experimental and Numerical Investigation

**DOI:** 10.3390/ma13071631

**Published:** 2020-04-01

**Authors:** Czesław Bywalski, Maciej Kaźmierowski, Mieczysław Kamiński, Michał Drzazga

**Affiliations:** Faculty of Civil Engineering, Wroclaw University of Science and Technology, Wybrzeże Wyspiańskiego 27, 50-370 Wrocław, Poland; maciej.kazmierowski@pwr.edu.pl (M.K.); mieczyslaw.kaminski@pwr.edu.pl (M.K.); michal.drzazga@pwr.edu.pl (M.D.)

**Keywords:** high-strength concrete, steel fibres, flexural tensile strength, fracture energy, numerical analysis

## Abstract

The paper presents the results of tests for flexural tensile strength (f_ct,fl_) and fracture energy (G_f_) in a three-point bending test of prismatic beams with notches, which were made of steel fibre reinforced high-strength concrete (SFRHSC). The registration of the conventional force–displacement (F–δ) relationship and unconventional force-crack tip opening displacement (CTOD) relationship was made. On the basis of the obtained test results, estimations of parameters f_ct,fl_ and G_f_ in the function of fibre reinforcement ratio were carried out. The obtained results were applied to building and validating a numerical model with the use of the finite element method (FEM). A non-linear concrete damaged plasticity model CDP was used for the description of the concrete. The obtained FEM results were compared with the experimental ones that were based on the assumed criteria. The usefulness of the flexural tensile strength and fracture energy parameters for defining the linear form of weakening of the SFRHSC material under tension, was confirmed. Own equations for estimating the flexural tensile strength and fracture energy of SFRHSC, as well as for approximating deflections (δ) of SFRHSC beams as the function of crack tip opening displacement (CTOD) instead of crack mouth opening displacement (CMOD), were proposed.

## 1. Introduction

Steel fibre reinforced high-strength concrete (SFRHSC), in comparison with plain high-strength concrete, is marked by a more quasi-plastic character and increased resistance to cracking on bending [1,2]. The following should be considered to be the most important parameters of dispersed reinforcement structure, which can inhibit cracking in the cement matrix: volume of fibres in the composite (V_f_) [3], slenderness of fibres (λ) [4,5], fibre material characteristics, spatial distribution of fibres in concrete [6], as well as adhesion of fibres to cement matrix, resulting from mechanical anchorage, adhesion, and friction [7]. The addition of steel fibres to concrete might improve some of its mechanical parameters, including compressive strength (f_c_) [8,9] and the modulus of elasticity (E_c_) [3,8,10]. The use of steel fibres in concrete also significantly increases its flexural tensile strength (f_ct,fl_) [3,4,9,11]. This improvement is insignificant in the range of proportional strains and clear after they are exceeded, both before reaching the breaking load value and after exceeding it. Knowing the mechanical response of the structure under loading conditions is crucial for the SFRHSC beams in bending.

**Figure 1:**
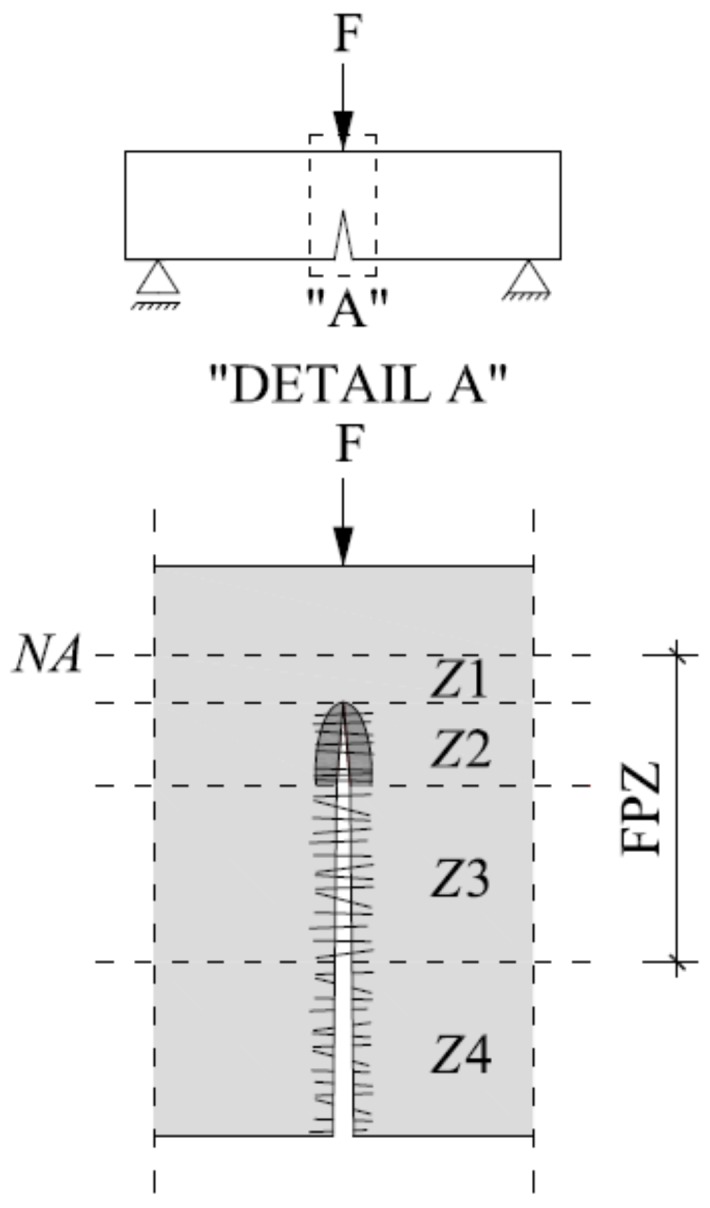
Cross-section of the three-point bending steel fibre reinforced concrete (SFRC) or steel fibre reinforced high-strength concrete (SFRHSC) beam before failure.

The behaviour of steel fibre reinforced concrete (SFRC) or SFRHSC beam in three-point bending test can be presented while using Tlemat’s proposal [7], which assumes a four-phase scope of work. Figure 1 shows the cross section of SFRC or SFRHSC beam before failure. Particular phases, depending on the load level, differ in the idealized distribution of stress blocks that result from the development of cracks in the fracture process zone (FPZ). The individual steps can be described, as follows: Phase 1—linear-elastic relationship between force (F) and deflection, no cracking in the zone of tensile stresses (zone Z1), slight influence of the slenderness of fibres (λ) on the value of the transferred load (F), maximum value of tensile stress being dependent on the concrete and volume of fibres in the composite (V_f_) [12]; Phase 2—the initiation of the micro-cracking process (0.1–0.2 mm), forces that result from the load are taken over by dispersed reinforcement in the zone Z2, the beginning of stress reduction in the FPZ, slight development of the fibre-pulling out process after crossing the limit load, change of the linear-elastic force–deflection relationship into the elastic-plastic relationship; Phase 3—further propagation of the cracking, development of fibre-pulling out process (zone Z3), continuation of force transmission by the fibres (zone Z2), raising the position of the neutral axis (NA); and, Phase 4—negligible transmission of forces by fibres (most of the fibres pull out from the cement matrix, zone Z4), no tensile stresses in the zone Z4, further possibility of load transfer provided by fibres in the zones Z2 and Z3 until the cracking reaches the neutral axis (NA).

Despite numerous studies, the method of testing and assessment of SFRHSC properties after cracking is still under discussion [11,13]. Technical committees propose various methods for determining the fracture energy and residual stresses. The lack of full standardization hinders the design and full use of this type of material in the construction industry. Therefore, there is a well-founded need for research in this direction. Differentiation applies to: loading method (three- or four-point bending test), shape and geometry of the researched element (beams, circular plates, cubes, etc.), or measured values (deflections, CMOD, CTOD).

The fracture resistance of SFRHSC is determined on the basis of standardized experimental tests [14]. According to [15], force–deflection (F–δ) or force-crack mouth opening displacement (F–CMOD) relationship in a three-point bending test are recorded. F–CMOD relationship analysis is widely used. It is performed by means of a clip gauge extensometer or a linear variable differential transformer (LVDT) [15]. The Italian National Research Council [16] recommends assessing the properties after cracking of steel fibre reinforced concrete based on the F–CTOD relationship. The analysis of the F–CTOD relationship is more experimentally difficult to perform as compared to F–CMOD. However, registering the force-width relationship of a crack opening is valuable from the point of view of conducting numerical analyses and cracking energy. Namely, it enables the direct comparison of experimental and numerical results and definition of a numerical material model (e.g., determination of the allowable crack width with tension in the concrete damaged plasticity model). In turn, Bencardino [14] showed that an assessment of fracture resistance could be carried out on the basis of force-crack tip opening displacement relationship (F–CTOD). Bencardino recorded F–CTOD and F–CMOD relationships for HSC and SFRHSC beams at the same time. Almost identical shape and size of F–CTOD and for F–CMOD curves could be observed. However, research of Bencardino must be continued to generalize the conclusions. Taking the above into account, the results of tests of F–CTOD relationship are also presented in the paper. In this way, the database of literature related to F–CTOD relationships has been extended. It also allowed for proposing equations for the approximation of SFRHSC beams deflections in CTOD function instead of CMOD [15].

The purpose of the article is to assess the flexural tensile strength (f_ct,fl_) and fracture energy (G_f_) of high-performance concrete with the addition of steel fibres (SFRHSC). The above parameters are essential when designing SFRHSC structural elements. The fracture energy of the SFRHSC composites is determined on the basis of standardized experimental tests [15]. The F–δ or F–CMOD relationships for three-point bending beams are recorded. Experimental tests were carried out in accordance with standard [15], and extended by registering the F–CTOD relationships, in order to achieve the above objectives. Additionally, the estimation of f_ct,fl_ and G_f_ quantities as a function of fibre reinforcement degree was made, and equations were developed, which contribute to the extension of standard records [15]; the standard currently allow an approximation of deflections of steel fibre reinforced concrete beams only as a function of CMOD. The obtained experimental results of the beams were compared with the numerical results (finite element method (FEM)) in quantitative and qualitative terms in order to assess the usefulness of the parameters of flexural tensile strength (f_ct,fl_) and fracture energy (G_f_), while also defining the linear form of weakening of the SFRHSC material under tension. The knowledge of the mechanical response of the structure under load is essential from the designer’s point of view concerning bending beams.

## 2. Experimental Research

### 2.1. Materials and Methods

The scope of research included making three series of composites (A, B, and C), which were differentiated by the content of steel fibres. Series A (control) did not contain any fibres; Series B contained 1.0% steel fibres (78 kg/m^3^); and, Series C included 1.5% steel fibres (118 kg/m^3^). The scope of tests and sample specifications are presented in Table 1. The samples used for strength tests were cured and protected according to [17].

The concrete mix was designed for the strength class C55/67 [18]. The following components were used in order to produce the concrete mixture: portland cement CEM-I 52.5N, natural washed aggregate with a fraction of 0–2 mm, broken granite aggregate with fractions 2–8 mm and 8–16 mm, polymer superplasticizer (Sika ViscoCrete 5-600 for high-performance concrete; properties: strong liquefaction (class S4/S5), no chlorides, increased early, and final strength of concrete mix, density 1.07 kg/dm^3^, PH 4.4 ± 1,0), and fly ash from hard coal (specific surface—3610 cm^2^/g; penetrability for the sieve with mesh 0.045 mm—38.50% [19]; mineralogical composition: SO_3_—0.38%, CaO—2.44%, SiO_2_—51.72%, Al_2_O_3_—25.15%, and Fe_2_O_3_—5.21%). The water-cement ratio of the mixture was 0.3 (w/c = 0.3). Straight steel fibres with a length of 20 mm (l) and a diameter of 0.3 mm (d) were added to the concrete samples, series B and C. The tensile strength of the fibres was 2000 MPa, while the Young’s modulus was 200 GPa. In order to obtain uniform consistency of concrete mix for all series, while maintaining a constant value of the w/c coefficient, test samples were prepared on the basis of which the optimal superplasticizer content was determined in individual composite series (A, B, and C). The consistency test was carried out using the drop cone method [20]. The indicator of the degree of mix liquidity ranged from 155 to 165 mm (consistency class S3/S4 [20]). Table 2 presents the composition of the concrete mix for individual series. All the components of the mixture were dosed by weight with an accuracy of 1% (without steel fibres and superplasticizer). The volume of each series of concrete mix was 453.2 dm^3^ (in accordance with the assumed programme of research with a wider scope). The order of dispensing the ingredients was as follows: the aggregate was mixed first, and then cement and fly ash were added. After thorough mixing, water was added, followed by a superplasticizer. Steel fibres were the last ingredient added to the mix. The total mixing time was 490 s. The concrete preparation equipment included: mixers to make trial batches, electronic scales for dosing the components of the concrete mix with an accuracy of 5 g and 0.1 g, a plate mixer (concrete plant), containers for transporting concrete, and a vibration table for compacting the concrete mix. The steel fibres and superplasticizer were dosed by weight with an accuracy of 0.1 g. Other components of the mixture were dosed with an accuracy of 5 g.

Experimental studies were carried out to determine the flexural tensile strength of HSC and SFRHSC, as well as their proportionality limit (f_ct,fl,L_) and the behaviour of beams in conditions of the exceeded critical limit load. This is why parallel relationships: force–deflection (F–δ) and force-crack tip opening displacement (F–CTOD) were observed. Characteristics of the tested composites, compressive strength (f_c_), and modulus of elasticity (E_c_) tests were carried out, owing to the need to obtain more accurate material. The obtained results were used for numerical analyses of HSC and SFRHSC beams.

The testing of the flexural tensile strength was carried out in accordance with [15]. During the study, parallel relationships: force–deflection (F–δ) and force-crack tip opening displacement (F–CTOD) were recorded. The adopted research program aimed, among others, at comparing these relationships.

**Figure 2:**
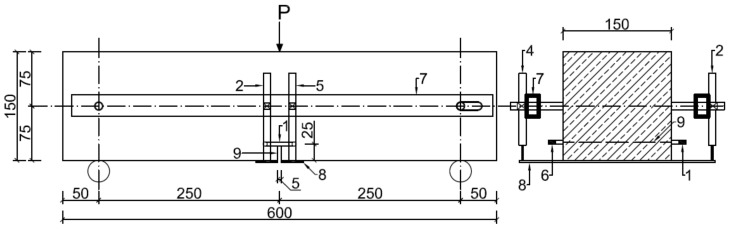
Test setup project: 1 and 6—sensors for measuring notch opening width (CTOD), 2–5—sensors for measuring deflection measurement (δ), 7—aluminum profile, 8—aluminum flat bar, and 9—notch.

**Figure 3:**
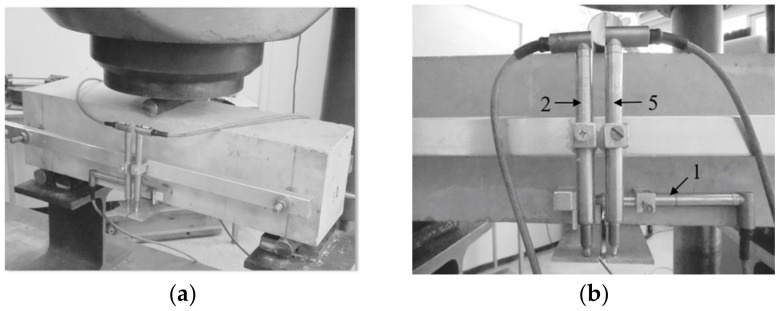
Flexural tensile strength test (f_ct,fl_): (**a**) C2 beam; (**b**) location of sensors for measuring deflections (No. 2 and 5) and notch opening (No. 1) in C1 beam.

Flexural tensile strength (f_ct,fl_) and the corresponding limit of proportionality (f_ct,fl,L_) were determined according to dependence (1), taking, for calculations, the limit load (F_max_) and the load corresponding to deflection (F_0.05_) respectively [15]. Figure 2 shows the test setup project. Figure 3a shows the beam prepared for testing, and Figure 3b depicts the detailed arrangement of the sensors for the measurement of top notch opening (CTOD) and deflection (δ).

(1) $$\sigma_{ct,fl}=\frac{3F_{i}l}{2bh_{sp}^{2}},$$

where:

l—beam span;
b—beam width; and,
h_sp_—beam height less notch height.

Measurement data CTOD and δ were recorded by means of extensometers with a range of 10 mm and accuracy of 0.001 mm, which were placed symmetrically along the longitudinal axis of the beam. The testing of beams was carried out at the ZD100 strength testing station (VEB Werkstoffprufmaschinen, Leipzig, Germany) with the computerized recording of synchronized results (time, F and δ). The frequency of records was 10 Hz.

Crack tip opening displacement (CTOD) means the width of a crack at the top of the technological notch. Figure 2 and Figure 3 indicate the location of the crack opening measurement (sensor No. 1). The height of the technological notch is 25 ± 1 mm, while the width is 4.98 mm (<5 mm). These values were adopted in accordance with the standard for testing fibre reinforced concrete [15]. The cuts were made while using a power saw with a guide, after prior marking. The dimensions of the technological notch were controlled with an electronic caliper.

The compressive strength tests (f_c_) were carried out in accordance with the standard [21] and the modulus of elasticity was measured according [22].

All of the tests were carried out after 28 days from preparing the composites.

### 2.2. Experimental Results and Discussion

#### 2.2.1. Results of Tests for Mechanical Features

The results of flexural tensile strength tests (f_ct,fl_), limit of proportionality (f_ct,fl,L_), compressive strength (f_c_) and the modulus of elasticity (E_c_) with the arithmetic mean of results x, and standard deviation for each series (s) are presented in Table 3.

The average density of composites (ρ) in each series was: A—2389 kg/m^3^, B—2493 kg/m^3^, and C—2522 kg/m^3^.

When analysing the results that are contained in Table 3, it can be seen that, with an increasing volume of steel fibres (V_f_) in the composite, the flexural tensile strength (f_ct,fl_) also significantly increases. For B series beams, the average increase in the value f_ct,fl_ when compared to that obtained for series A beams (control series) was 95.0%, and for C series beams 132.1%. It should be noted that the results of flexural tensile strength (f_ct,fl_) of SFRHSC beams can be burdened with a large spread of results (V = 20–30%) [23,24]. This might be due to, among other factors, the effect of the scale (different surfaces of the beams’ breakthrough) or the efficiency of anchoring the fibres in the cement matrix. The average value of stresses defining the conventional limit of proportionality of bending beams was for series B—6.14 MPa and for series C—7.01 MPa, while the coefficient of variation was 11.4% and 7.3%, respectively.

**Figure 4:**
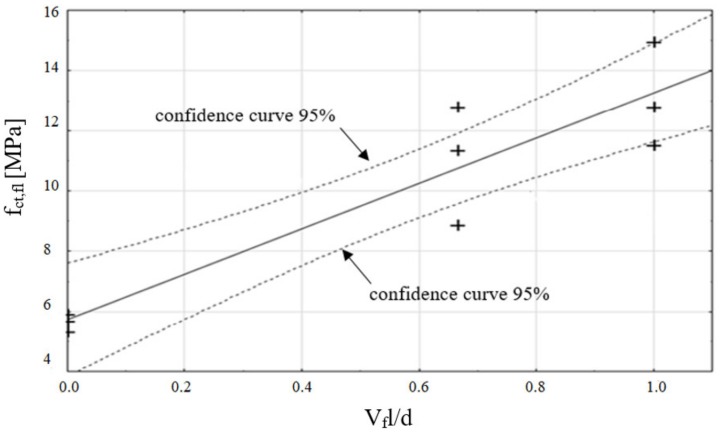
Flexural tensile strength results (f_ct,fl_), depending on the fibre reinforcement ratio (V_f_l/d), the regression curve, and 95% confidence curves.

Figure 4 presents the results of flexural tensile strength (f_ct,fl_), depending on the fibre reinforcement ratio (V_f_l/d) and a linear regression Equation (2) with the determination coefficient of R^2^ = 86%, which is a measure of the quality of model fit. The root from the variance of regression equation estimators was 1.15 for the directional coefficient and 0.80 for the free expression, respectively. It should be noted that a large confidence interval of the directional regression coefficient was obtained (2.72), owing to the small size of the sample (nine results). On the basis of the statistical test carried out (Student’s t-distribution), for the assumed level of significance α = 5%, the obtained regression coefficient of the model is statistically significant (p < α, where p is the probability of accepting the null hypothesis of the parameter).

(2) $$f_{ct,fl}=f_{\mathrm{ct},\mathrm{fl}}^{\prime }+7.54\left(\frac{V_{f}l}{d}\right),$$

where: $f_{ct,fl}^{\prime }$—the flexural tensile strength of HSC.

**Figure 5:**
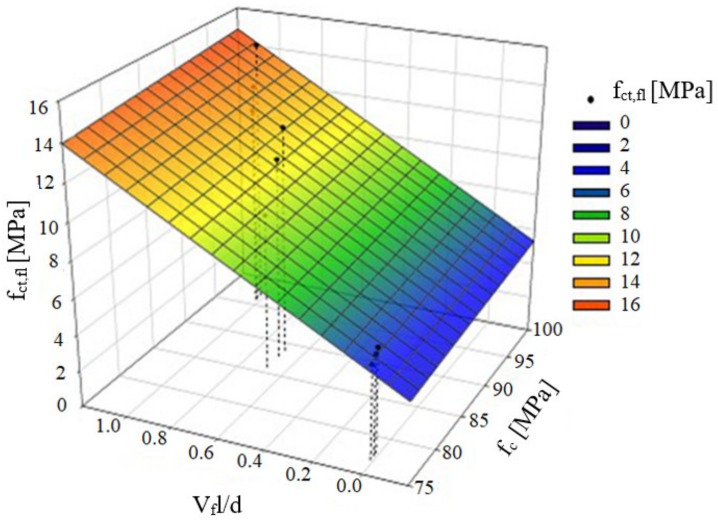
Flexural tensile strength results (f_ct,fl_) depending on compression strength (f_c_) and fibre reinforcement ratio (V_f_l/d).

As part of the analysis of the model’s fit to the empirical data, the authors also considered various parametric models with two explanatory variables in the form of compressive strength (f_c_) and fibre reinforcement (V_f_l/d), which are available in the literature on the subject [3,4,25,26]. Consequently, a non-linear regression Equation (3) with a coefficient R^2^ = 86.4% was obtained. The results of flexural tensile strength (f_ct,fl_), depending on the compressive strength (f_c_) and fibre reinforcement ratio (V_f_l/d), as well as a graphical presentation of the Equation (3) are shown in Figure 5. It should be noted that the regression equation applies to the range of variables under consideration (compressive strength 76–96 MPa; slenderness of fibres 66.7; and, fibre content by volume 0; 1.0; 1.5%).

(3) $$f_{ct,fl}=0.66\sqrt{f_{c}}+\frac{6.88V_{f}l}{d}$$

The roots of the compressive strength estimate and the fibre reinforcement ratio of model (3) were 0.09 and 1.21, respectively. Statistically significant structural parameters of the model (p < α) were obtained on the basis of the Student’s *t*-test, for the assumed level of significance α = 5%.

Volumetric addition of steel fibres (V_f_) in series B (1.0%) and C (1.5%) resulted in an increase in the compression strength (f_c_) in relation to series A (control) by 12.9% and 24.3%, respectively. The obtained increase in compressive strength (f_c_) of SFRHSC concretes is confirmed in the scientific literature on the subject [3,4,27], which reports, for the addition of fibres (V_f_) in the range of 0–1.5%, an increase in compressive strength in the case of SFRHSC from 10% to 20%, as compared with HSC.

The presence of steel fibres in the composite also increased the value of the modulus of elasticity (E_c_). For the B and C series, the increase in the modulus of elasticity in relation to the reference series (series A) was 15.0% and 17.0%, respectively. It was observed that the increase in fibre content from 1.0% to 1.5% (V_f_) in the composite (C series results) resulted in only a slight increase in the modulus of elasticity (by 2.0%). A similar increase in the modulus of elasticity (E_c_) of SFRHSC in relation to HSC can be found in [8,28,29]. The increase ranges from 7.0% to 27.0%, for the addition of steel fibres in the range of 0–1.5% in the composite. It should be noted that, along with the increase in the content of steel fibres in the composite (V_f_), the compressive strength (f_c_) [10,30] and modulus of elasticity (E_c_) [31,32] are not always significantly increased.

#### 2.2.2. F–δ and F–CTOD Relationship

**Figure 6:**
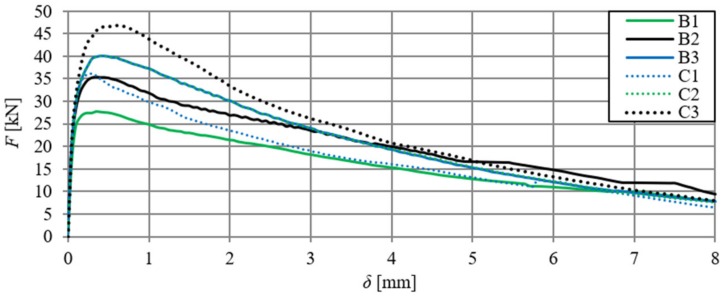
Force–deflection relationships for SFRHSC beams (B and C series, curve C2 coincides with curve B3).

**Figure 7:**
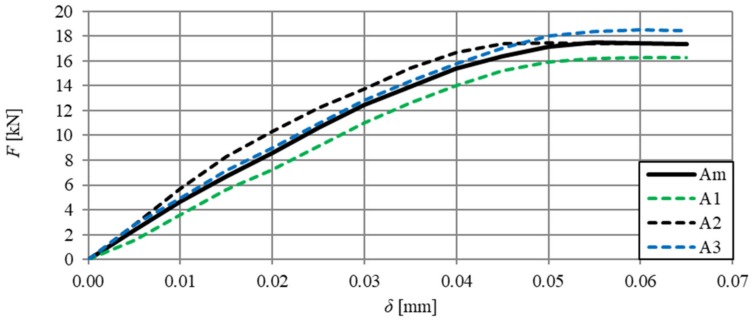
Force–deflection relationships for HSC beams (series A) and curve F–δ obtained on the basis of the arithmetic mean of results (continuous line).

**Figure 8:**
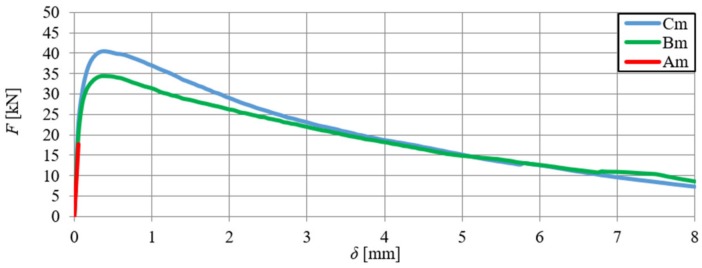
Force–deflection relationships obtained on the basis of the arithmetic mean of the results for beams from series A, B, and C.

**Figure 9:**
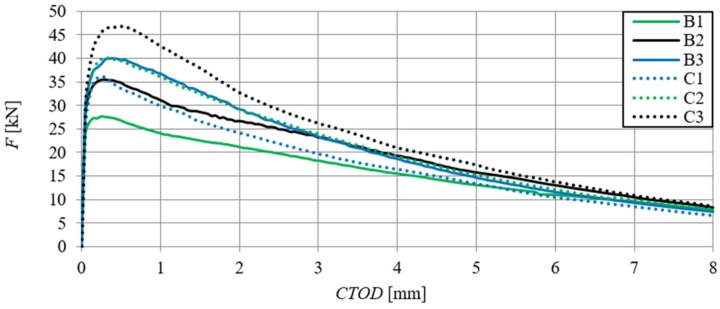
Force-crack tip opening displacement relationships of SFRHSC beams (B and C series).

**Figure 10:**
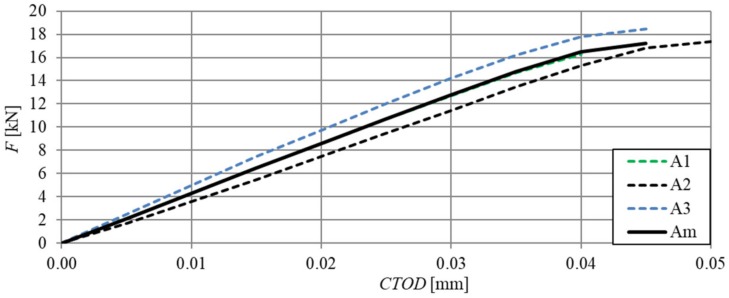
Force-crack tip opening displacement relationships of HSC beams (series A) and the F–CTOD curve obtained on the basis of the arithmetic mean of the results (continuous line, curve A1 coincides with curve Am).

**Figure 11:**
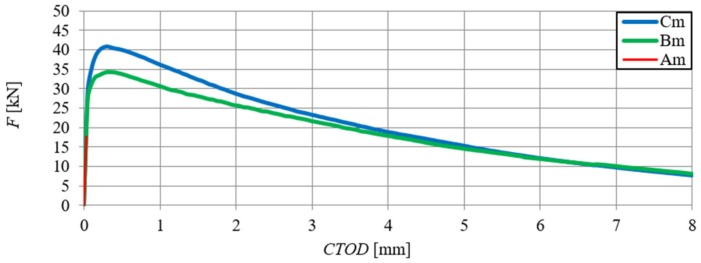
Force-crack tip opening displacement relationships obtained on the basis of the arithmetic mean of the results for beams in series A, B and C.

Figure 6 shows the relationships of force–deflection (F–δ), in particular SFRHSC beams, and in Figure 7 the same relationship can be seen for HSC beams. The deflection values (δ) were the arithmetic mean of the sensor readings (Nos. 2–5, according to Figure 2). The force–deflection relationships that were obtained on the basis of the results of the arithmetic mean of beams in particular series (A, B and C) are shown in Figure 7 and Figure 8 (Figure 7—due to the smaller range of HSC beams deformation in relation to SFRHSC beams). The second type of relationship, namely force-crack tip opening displacement (F–CTOD), which was measured during the HSC and SFRHSC beam tests, is shown in Figure 9 and Figure 10, respectively. The values of individual beams CTOD placed on the graph are the mean of the sensors nos. 1 and 6 (see Figure 2). The F–CTOD relationships that were obtained on the basis of the arithmetic mean of the results in particular series are shown in Figure 11 (series A, B, and C) and Figure 10 (series A only).

**Figure 12:**
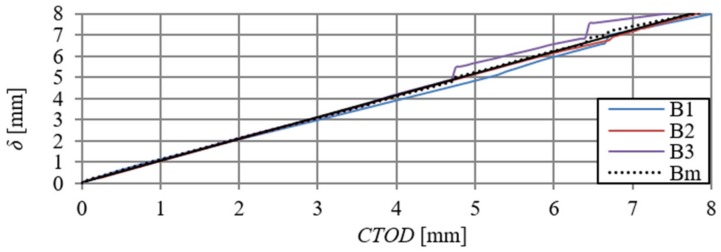
Deflections–crack tip opening displacement relationship of B-series beams and δ–CTOD curve obtained on the basis of the arithmetic mean of results (Bm).

**Figure 13:**
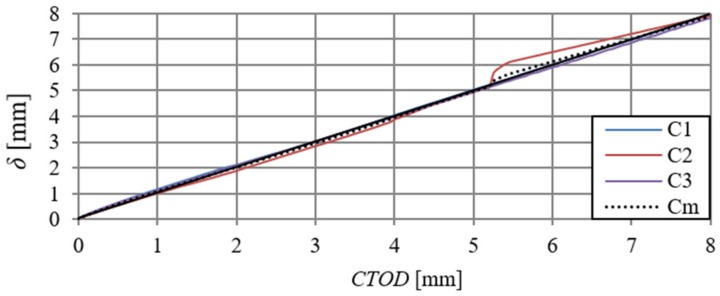
Deflection–crack tip opening displacement relationships of C-series beams and the relationship δ–CTOD obtained on the basis of the arithmetic mean of results (Cm).

When analysing Figure 6 and Figure 9, one can observe minimal differences in the shape and size of curves describing F–δ and F–CTOD relationships (curves overlap). Figure 12 presents deflections of the B series beams, depending on the crack tip opening displacement (CTOD) and the δ–CTOD relationship obtained on the basis of the arithmetic mean of the series results. Analogous results are presented in Figure 13 for C-series beams. The δ–CTOD relationships that were obtained on the basis of the arithmetic mean of series B and C results can be approximated by linear Equations (4) and (5) (R^2^ = 99%), respectively. These equations could be a contribution to the extension of the standard records [15], which presently allow for an approximation of deflections (δ) of beams that were modified with steel fibres only as a function of crack mouth opening displacement (CMOD).

(4) δ=1.03CTOD+a,

where: a—constant term; a = 0.02, if CTOD > 0; a = 0, if CTOD = 0.

(5) δ=0.99CTOD+b,

where: b—constant term; b = 0.05, if CTOD > 0; b = 0, if CTOD = 0.

It should be noted that the regression Equations (4) and (5) were determined for specific laboratory and material conditions of the composite. In addition, they relate to the considered range of variables, and they should be verified in the future on a larger number of research results.

**Figure 14:**
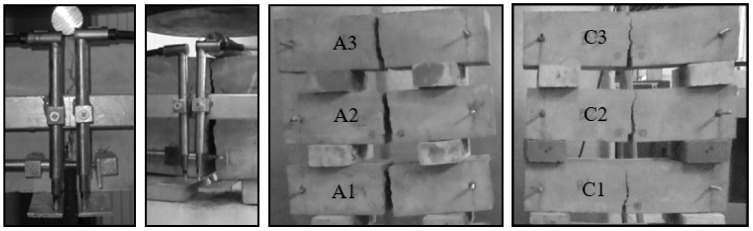
Damaged beams; from left: beam C2; beam A1 (no fibres); series A; series C.

Figure 14 shows the damaged HSC and SFRHSC beams (owing to a similar failure mode of beams, only C series is presented). The presence of fibres affects the destruction characteristics of SFRHSC beams, which do not suddenly divide in contrast to HSC ones. After they are “destroyed”, SFRHSC beams are able to carry a set load (usually smaller than the breaking load) until the broken parts are “separated”.

#### 2.2.3. Fracture energy of HSC and SFRHSC

The fracture energy, which is marked by the range of post-critical stress–elongation of concrete dependence (σ–w), can be treated according to Hilleborg’s proposal and the RILEM technical committee [33,34], as the material constant of the composite (other methods for calculating fracture energy are also applicable [35]). It determines the amount of energy that is required to produce a crack with a unit area that can be calculated while using the Equation (6).

(6) $$G_{f}=\frac{1}{bh_{sp}}\left[\int_{0}^{\delta_{u}}F\left(\delta \right)d\delta +m\left(1-\alpha^{2}\right)g\delta_{u}\right],$$

where: m—beam mass; g—gravitational acceleration; δ_u_—maximum deflection recorded during the test; α = 1 − L/2l; L—beam length; b, h_sp_, l—explanations identical to Equation (1).

**Figure 15:**
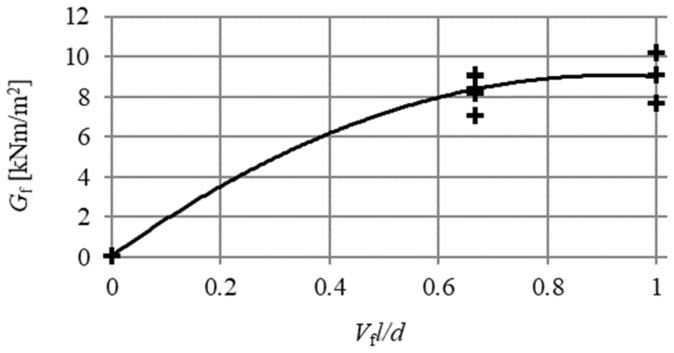
HSC and SFRHSC beams’ fracture energy results (G_f_) depending on fibre reinforcement ratio (V_f_l/d) and polynomial regression Equation (7).

Table 4 shows the results of the fracture energy for particular beams, along with the arithmetic mean (x) and the standard deviation (s) for each series. Figure 15 shows the influence of fibre reinforcement ratio (V_f_l/d) upon the value of beam fracture energy (G_f_) and the polynomial regression Equation (7) with the coefficient R^2^ = 96% (p < α). The roots from the estimators’ variance of the polynomial regression model (7) were, respectively: 3.15; 3.21; and, 0.57. On the basis of the Student’s *t*-test, for the assumed level of significance α = 5%, the obtained regression coefficients for the model are statistically significant (p < α).

(7) $$G_{f}=-10.72{\left(\frac{V_{f}l}{d}\right)}^{2}+19.68\left(\frac{V_{f}l}{d}\right)+0.04.$$

It should be noted that regression Equation (7) was determined for specific material conditions of the composite, and it applies to the considered range of variables (V_f_, l, d and type of fibre). It should be remembered that the length, diameter, type, and volume of fibre strongly influence the fracture energy.

## 3. Numerical Analysis of Beams

### 3.1. Assumptions for the Numerical Model and Geometrical Model

Numerical analyses of SFRHSC beams were performed to assess the usefulness of the flexural tensile strength and fracture energy parameters for defining the linear form of weakening of the SFRHSC material under tension, using a concrete damaged plasticity (CDP) model. To this end, quantitative and qualitative analyses were performed. Numerical calculations of HSC and SFRHSC beams were made in the Abaqus/Standard [36] program while using the CDP model.

The following assumptions were made in the numerical calculations: the influence of steel fibres on the behaviour of the composite was taken into account on the basis of calculated composite fracture energy (*G*_f_); attention was focused on physical nonlinearity describing HSC and SFRHSC; nonlinear effects were included using the Newton–Raphson increment-iterative method; and, the calculations were performed in the plane state of strain.

**Figure 16:**
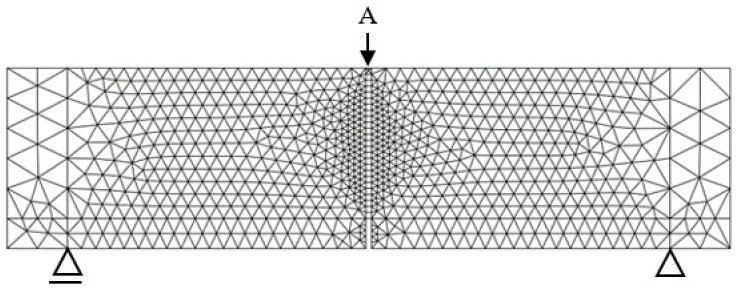
Finite-element mesh and boundary conditions for the beam used in numerical calculations.

The discretization of the beam in the numerical model was performed with the use of 1539 finite elements. Plane stress elements were used, four-node with reduced integration (CPS4R), located above the notch (in the place of stress concentration–crack propagation), with a mesh size of 5 mm (dimension determined by the notch width), and three-node (CPS3), located on the remaining area of variable dimensions (5–25 mm). The mesh of finite elements and boundary conditions for the beam, which were used in numerical calculations, are shown in Figure 16. Load of the beam was carried out in a kinematic way through the task of displacement (Figure 16, point A), changeable in time. The displacement point in the model was located in accordance with the place of force effects during experimental beam testing. The displacement value was δ = 8 mm for SFRHSC beams and δ = 0.1 mm for HSC beams. The size of the load increment in the range and 0.001,1E−10 the maximum number of load steps of 5000 were assumed.

The use in numerical simulations, in the non-linear σ_t_–ε_t_ relationship (weakening of the material), leads to highly sensitive results for the discretization of finite elements [36,37] (ambiguous response at the level of structure depends on the deformation location zone); hence, alternatively, the form of material weakening can be described with the use of fracture energy (G_f_). In this case, the brittle damage is described by the dependence $\bar{\sigma_{t}}-u_{t}^{-pl}$ ($\bar{\sigma_{t}}$—effective stress), instead of $\bar{\sigma_{t}}-\varepsilon_{t}^{-pl}$, which is initiated when the crack (associated with the deformation location) begins to propagate, after the material reaches stress that is equal to the axial tensile strength (σ_t0_). With the opening of the crack (the displacement increases), the tensile stress decreases up to the zero value, which corresponds to the maximum displacement ($u_{t,max}^{-pl}$). The dissipation of energy in the creation of cracks with a unitary field is expressed by the formula:

(8) $$G_{f}=\int_{0}^{u_{t,max}^{-pl}}\bar{\sigma_{t}}du_{t,max}^{-pl}.$$

The above dependence shows that the energy that is released during cracking (G_f_) is equal to the value of the area under the curve $\bar{\sigma_{t}}-u_{t}^{-pl}$, and the curves themselves may assume different forms of weakening, e.g., linear or exponential.

The force-displacement at the construction level does not depend on the finite element discretization given the above relationships [37]. Due to its simplicity, this modelling method is often used in commercial FEM programs [38]. The shortcomings of the method involve limiting the location zone to one row of elements and reducing its width along with the compaction of the mesh, instead of remaining constant. It should be noted that the above limitations do not affect the mathematical model of non-linear deformations related to cracking or damage [36,37]. An improved continuous medium model can be used in order to eliminate irregularities in the mathematical model for weakened material (getting rid of the pathological dependence of the discrete solution on the mesh) [39,40].

### 3.2. Material Model

For the numerical analysis of HSC and SFRHSC beams, the default values of the model parameters were adopted, defining its operation in a complex stress state (β, є, f, K_c_, μ) [36], and they are shown in Table 5. It has been assumed that according to the standard [18], the Poisson’s ratio of uncracked concrete equals 0.2.

In Table 5: β—the pitch of the hyperbolic asymptote of the Drucker–Prager surface to the hydrostatic axis, as measured in the meridional plane; є—eccentricity of the plastic potential, being a small positive value characterizing the speed at which the hyperbole of the plastic potential is approaching its asymptote; f—a number defining the quotient of the limit compressive stress in the biaxial state to the limit compressive stress in the uniaxial state; K_c_—a parameter defining the shape of the surface of the plastic potential on the deviatorial plane, which depends on the third invariant of the stress state; and, μ—a viscous parameter, allowing to barely exceed the surface of plastic potential, in some sufficiently small steps of the task.

Density (ρ), the modulus of elasticity (E_cm_), and compressive strength (f_cm_) of individual composite series were adopted on the basis of experimental tests (see point 3.1). In identifying the parameters in the uniaxial state of compression (σ_c_–ε) of A, B, and C beams, it was assumed that concrete behaves in a linear elastic manner to linear stress 0.6 f_cm_ [41], and it is then strengthened to the stresses equal to f_cm_, which corresponds to strains that are equal to ε_c,in_ = 1.5‰.

**Figure 17:**
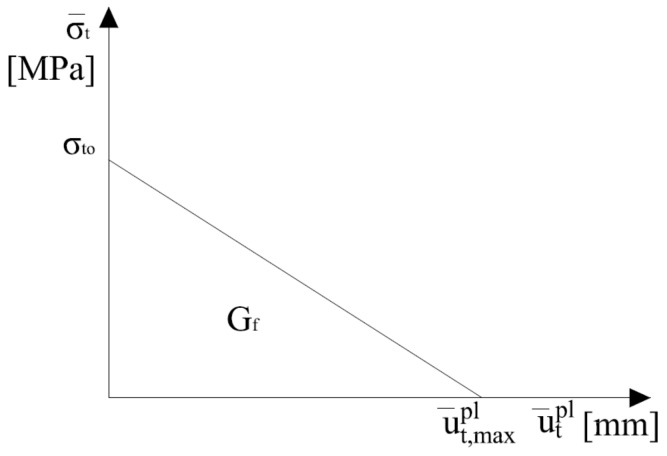
Linear weakening during tension.

The material degradation associated with crushing of concrete was ignored (d_c_ = 0), owing to the obtained failure mode of beams triggered off by cracking during tension. By tension, linear form of composite weakening ($\sigma_{t}^{-}-u_{t}^{-pl}$) was assumed, as in Figure 17. In this case, the constitutive parameter defining destruction during tension, destructive displacement ($u_{t}^{-pl}$), was determined according to the relationship [36]:

(9) $$u_{t,max}^{-pl}=\frac{2G_{f}}{\sigma_{t0}}.$$

For calculations $u_{t,max}^{-pl}$, the average values of fracture energy of individual series of beams (A, B, and C) were taken; they come from Table 4. Owing to the lack of own tests of axial tensile strength of SFRHSC samples, the parameter σ_t0_ was determined on the basis of the results of the proportionality limit (f_ct,fl,L_) [15] and then, owing to the complexity of the problem of determining the relationship σ_t0_–f_ct,fl,L_ as well as the small number of experimental studies in this respect, a simplification was made that consisted of accepting the estimate based on the Raphael dependency [42], based against on Navier’s hypothesis (dependence obtained on the basis of tests for concrete without fibres):

(10) $$\sigma_{t0}=0.75f_{ct,fl,L}.$$

In the case of HSC beams, the parameter *σ*_t0_ was calculated in an analogous way, with flexural tensile strength (f_ct,fl_). An arbitrary stress of 0.01 *σ*_t0_ was accepted and material degradation of d_t_ = 0.95 was assumed in order to avoid loss of convergence due to zero stress and stiffness. Table 6 presents details of the material parameters implemented for each series of beams.

### 3.3. Results of Numerical Analyses

**Figure 18:**
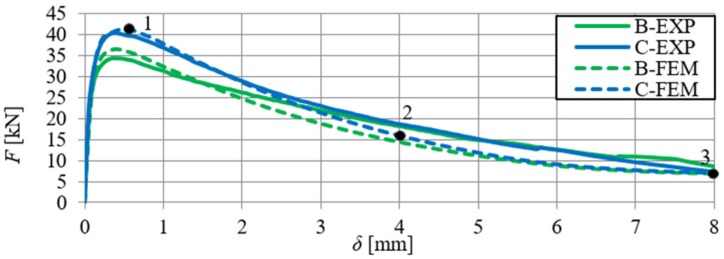
Comparison of numerical and experimental force–deflection relationship of SFRHSC beams in a three-point bending tests (B and C series).

**Figure 19:**
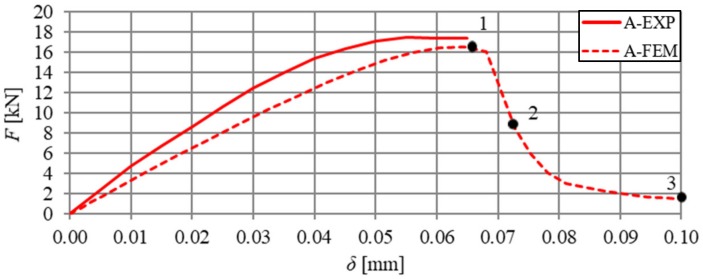
Comparison of numerical and experimental force–deflection relationship of HSC beams in three-point bending tests (series A).

**Figure 20:**
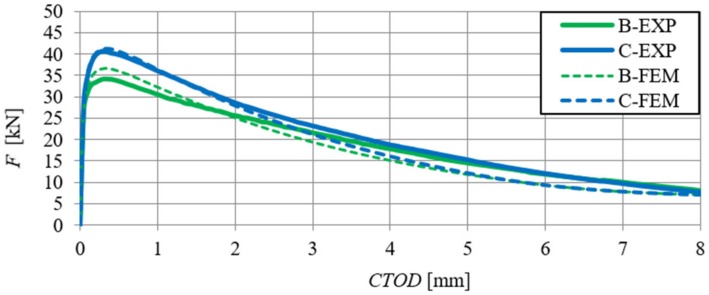
Comparison of numerical and experimental force-crack tip opening displacement relationship of SFRHSC beams in three-point bending tests (B and C series).

**Figure 21:**
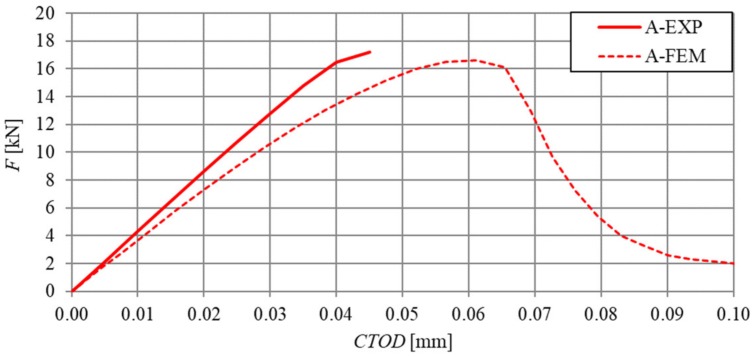
Comparison of numerical and experimental force-crack tip opening displacement relationship of HSC beams in three-point bending tests (series A).

A comparative analysis was carried out in order to verify the numerical model, which included the relationships F–δ and F–CTOD (quantitative analysis) and crack propagation (qualitative analysis). The analysis was performed on the basis of the arithmetic mean of beam results for each series. In Figure 18 and Figure 19, the static equilibrium path (F–δ) of the FEM model was compared with the experimental results that refer to the HSC and SFRHSC beams, respectively. In turn, numerical and experimental F–CTOD curves were compared in Figure 20 and Figure 21.

The comparative analysis of the relationships F–δ and F–CTOD confirms reliable compatibility of the obtained results of experimental and numerical test on SFRHSC beams. Compatibility can be observed both for the limit load (load-bearing capacity) and the behaviour of beams in the beyond-elastic range. In the case of HSC beams, there are some differences in the elastic work of the structure. This could be due to the influence of the test methodology on the value of the modulus of elasticity (E_c_). The obtained numerical results confirm the overall compatibility of the computational model with assumptions regarding the material strength hypothesis. The differences between the experimental stiffness of the beams and the stiffness of the beams in the numerical model are also illustrated in the graphs. It confirms the correct selection of the degradation variable in the concrete model d_t_ and the right assumption of simulating the fibres in the material based on the fracture energy.

**Figure 22:**
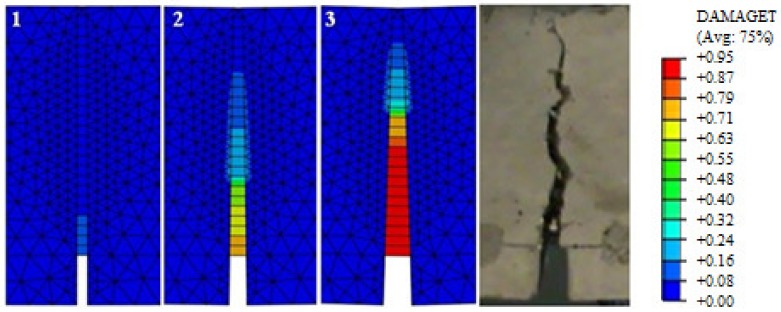
Image of finite element method (FEM) destruction corresponding to the characteristic points of the static equilibrium path (see Figure 18) of SFRHSC beams (series C); the final image of the destruction of the tested C2 beam and the legend for the material damage map.

**Figure 23:**
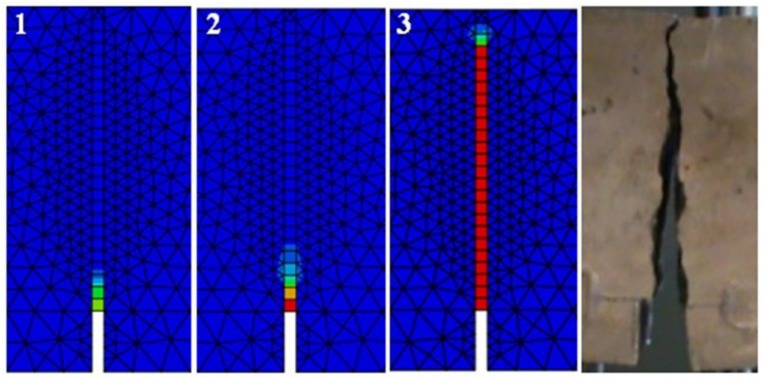
Image of FEM destruction corresponding to the characteristic points of the static equilibrium path (see Figure 19) of the HSC beams (series A) and the final picture of destruction of the tested A1 beam.

The comparative analysis of the cracking in the beams, for the clarity of the argument, was limited to two cases: series A and C (the largest volume share of fibres in the composite and their absence). The analysis was made on the basis of damage map comparison, being defined by changes in the value of parameter DAMAGET (d_t_ degradation of stiffness illustrating the destruction of material). The damage maps were prepared for the characteristic points of the static balance paths of HSC and SFRHSC beams (points 1, 2, and 3 marked in Figure 18 and Figure 19), which are shown in Figure 22 and Figure 23, respectively. The FEM images of the damaged HSC and SFRHSC beams were compared to images that were obtained during the experiment (Figure 22 and Figure 23).

Since, in the CDP model, it is not possible to shape cracks in a discrete way, taking into account the chipping of the material, damage maps (DAMAGET) should be identified with the gradual exclusion of finite elements from cooperation. In this way, their specific ‘gluing’ and further participation in the transmission of deformations to adjacent elements takes place. This imperfection does not have a significant impact upon the behaviour of the entire research element (convergence of static equilibrium pathways) [43].

In numerical analyses, the obtained damage images qualitatively correspond to the crack propagation images, which were obtained during experimental studies (Figure 22—SFRHSC beam and Figure 23—HSC beam). Moreover, the analysis of damage maps that were defined by the parameter d_t_ made it possible to follow the process of crack formation and development with the increasing load of the model. For the SFRHSC beam model (C series), the development of the fracture process zone (crack range) was “smooth” (successive elimination of finite elements from cooperation), which should be identified as crack propagation due to gradual force transmission through the fibres and crack propagation was rapid and violent in the case of the HSC beam (A series).

## 4. Final Conclusions

On the basis of the presented research results, performed analyses, and literature review, the following conclusions were formulated:

– The tests that were carried out on beams have shown that together with the increase in the volumetric amount of fibres (V_f_) in the beam, flexural tensile strength increases considerably (f_ct,fl_).
– Laboratory tests carried out showed considerably higher (over two hundred times) fracture energy (G_f_) in the case of SFRHSC beam as opposed to HSC beams.
– The presence of fibres affects the destruction characteristics of SFRHSC beams, which do not suddenly divide in contrast to HSC ones. After they are “destroyed”, the SFRHSC beams are able to carry a set load (usually smaller than the breaking load) until the broken parts are “separated”.
– Regression Equations (2), (3), and (7) with determination coefficients R^2^ > 85% and statistically significant structural parameters were obtained as a result of statistical analysis of the test results (f_ct,fl_, f_c_ and G_f_). These equations can be used to estimate the flexural tensile strength (f_ct,fl_) and fracture energy (G_f_) for SFRHSC beams. In view of the sample size (≤9), the proposed regression equations should be verified in the future by performing experimental tests on a larger number of samples.
– For SFRHSC beams, the force–deflection (F–δ) and force-crack tip opening displacement (F–CTOD) relationships were almost identical (shape and area under the graph). The relationship between the deflection and crack tip opening displacement (δ–CTOD) obtained for the mean results of B- and C-series beams is described by linear regression Equations (4) and (5). These equations could be a contribution to the extension of the standard [15], which now allow for approximating the deflections (δ) of steel-fibre-modified beams only as a function of crack mouth opening displacement (CMOD).
– Numerical analysis of HSC and SFRHSC beams with the use of the CDP model showed high compliance with the experimental results in terms of quantity (conformity of the F–δ and F–CTOD relationships) and quality (image of the cracking).
– Minor differences between the results of laboratory tests and numerical analyses of HSC and SFRHSC beams confirm the reliability of the parameters adopted, experimentally and theoretically determined, and describing the elastic-plastic material model used in numerical simulations.
– The basic material parameter used in numerical simulations (CDP model), which is employed to describe the behaviour of SFRHSC beams in the beyond-elastic range is the fracture energy (G_f_), takes into account the presence of fibres in the composite. To determine the energy, it is necessary to know, for example, the relationship: F–δ, F–CMOD or F–CTOD.

## Figures and Tables

**Table 1:** Scope of tests and specification of samples.

Measured Parameter	Sample Type	Sample Dimensions (mm)	Series			Total
			A	B	C	
f_ct,fl_, f_ct,fl,L_	beam	150 × 150 × 600	3	3	3	9
f_c_	cube	150 × 150 × 150	3	3	3	9
E_c_	cylinder	150 × 300	5	5	5	15

**Table 2:** Concrete mixture composition.

Material		Series		
		A	B	C
Steel fibres	kg/m^3^	0	78	118
	% (V_f_) *	0	1.0	1.5
Superplasticizer	kg/m^3^	8.25	9.65	10.80
Cement-CEM I 52.5R	kg/m^3^	550		
Sand 0–2 mm	kg/m^3^	600		
Granite aggregate 2–8 mm	kg/m^3^	490		
Granite aggregate 8–16 mm	kg/m^3^	590		
Fly ash	kg/m^3^	30		
Coefficient w/c	-	0.30		

* V_f_ = W_f_/ρ_f_ (W_f_—fibre content in mass units in 1 m^3^; ρ_f_—fibre material density in kg/m^3^).

**Table 3:** Test results.

Series	f_ct,fl_ (MPa)			f_ct,fl,L_ (MPa)			f_c_ (MPa)			E_c_ (GPa)		
	Result	x	s	Result	x	s	Result	x	s	Result	x	s
A		5.64	0.30		-	-		76.91	0.47	34.50	35.68	1.64
	5.32			-			76.41			36.90		
	5.69			-			76.97			33.47		
	5.91			-			77.34			36.17		
										37.35		
B		11.00	1.99		6.14	0.70		86.84	1.52	40.35	41.08	1.21
	8.86			5.34			85.19			41.07		
	11.34			6.42			87.16			39.80		
	12.79			6.65			88.17			42.99		
										41.20		
C		13.09	1.74		7.01	0.51		95.56	0.44	37.77	41.76	2.36
	11.52			6.42			95.19			41.61		
	12.80			7.27			95.44			43.73		
	14.96			7.33			96.04			42.56		
										43.12		

**Table 4:** HSC and SFRHSC beams fracture energy results along with the arithmetic mean (x) and standard deviation (s) for each series (kNm/m^2^).

Beam	Result	x	s
A1	0.036	0.0412	0.004
A2	0.043		
A3	0.044		
B1	7.051	8.394	1.16
B2	9.033		
B3	9.098		
C1	7.688	8.996	1.26
C2	9.103		
C3	10.195		

**Table 5:** The default parameters of the concrete damaged plasticity (CDP) model in the complex stress state.

Name of Parameter	β	є	f	K_c_	μ
Value	36°	0.1	1.16	0.667	0

**Table 6:** Material constants adopted for the CDP model for different composite series.

	The Law of Strengthening in Compression		The Law of Weakening in Tension		
Series	σ_c_ (MPa)	$\varepsilon_{c}^{-in}\cdot 10^{-3}$	σ_t_ (MPa)	$u_{t}^{-pl}$ (mm)	d_t_
A	46.15	1.29	4.23	0	0
	76.91	1.50	0.042	0.04	0.95
B	52.10	1.27	4.60	0	0
	86.84	1.50	0.046	3.648	0.95
C	57.34	1.37	5.25	0	0
	95.56	1.50	0.053	3.424	0.95
